# Sense of agency may not improve recollection and familiarity in recognition memory

**DOI:** 10.1038/s41598-022-26210-1

**Published:** 2022-12-15

**Authors:** Nanami Tsuji, Shu Imaizumi

**Affiliations:** 1 Graduate School of Humanities and Sciences, Ochanomizu University, Tokyo, Japan; 2 Institute for Education and Human Development, Ochanomizu University, Tokyo, Japan

**Keywords:** Human behaviour, Cognitive neuroscience, Learning and memory

## Abstract

Sense of agency (SoA) is a feeling of controlling one’s own action. Recognition memory can improve for stimuli that involve SoA perhaps because of the self-reference effect. Recognition memory consists of *recollection* (i.e., detailed memory of stimuli) and *familiarity* (i.e., a feeling that stimuli are in memory). The self-reference effect is often observed in the recollection. Here, we investigated whether SoA particularly improves the recollection process. Participants pressed a key to produce an outcome (i.e., box movement followed by word presentation in Experiment 1 or word presentation in Experiment 2) and rated their SoA over the outcome. The outcome was spatially congruent or incongruent with the action. The participants learned the words intentionally (Experiment 1) or incidentally (Experiment 2). Performances of recollection and familiarity were assessed using the remember/know procedure. Our results suggest that the participants’ SoA was successfully manipulated. However, contrary to our hypothesis and previous findings, we found no effects of voluntary action and action–outcome congruence on recollection and familiarity processes of recognition memory, regardless of the latency of word presentation and learning strategies. Further studies are needed to replicate and elucidate the relationship between the SoA and recognition memory.

## Introduction

When students prepare for an important exam, how do they remember the words or images they need to memorize? They might repeat the words or recall the images while connecting them to the relevant information. Motor action is one of the effective ways of remembering. For example, research has shown that memory performance for enacted events (e.g., enactment following instructions) is better than for unenacted events, such as merely listening to instructions (i.e., the enactment effect^1^).

Even simple actions like pressing a key can improve memory. Previous studies have shown that recognition memory for visual stimuli is better when the stimuli prompt a Go response with a key press than when they prompt a No-Go response without a key press^2–5^. Enhanced memory performance in the Go condition can be explained by reduced performance in the No-Go condition, where attentional resources are more on the action (inhibitory) control than on encoding the stimulus features^2,3^. However, Yebra et al.^4^ suggested that motor action per se activates the locus coeruleus, a source of noradrenaline that enhances episodic memory encoding and improves recognition memory (i.e., action-induced memory enhancement). In contrast, Kinder and Buss^6^ and Shimane et al.^7^ observed an enhancement effect by employing a non-motor cognitive task (i.e., counting) to establish a baseline for memory performance in a motor task that required Go and No-Go responses. Performance was higher for the motor task than for the cognitive one, suggesting that engagement of motor system enhances memory regardless of motor execution or inhibition.

Few studies on the enactment effect and action-induced memory enhancement have focused on awareness and self-recognition associated with an action. However, these may play a role in the relationship between action and memory. One study found the enactment effect even in a patient with a loss of proprioception below the nose and suggested that the intention of action and motor commands for action could enhance memory^8^. Therefore, memory performance might also be related to awareness of one’s own actions.

Sense of agency (SoA) refers to a subjective feeling of controlling one’s own action^9^. One model suggested that the SoA emerges when an action outcome predicted by the internal forward model is spatiotemporally consistent with the actual outcome^10^. Spatial and temporal (in)congruence between the predicted and actual outcomes modulates the degree of SoA. For example, in a task where participants used a computer mouse to move a cursor toward a specific screen location, an angular bias and a delay in cursor movements induced spatiotemporal incongruence with the prediction, reducing SoA^11^. That finding has been replicated using other visuomotor tasks, such as joystick manipulation^12^ and a single key press^13^.

Hon and Yeo^14^ suggested that the recognition (i.e., hit rate) of word stimuli was better when participants experienced stronger SoA over the appearance of words initiated by their key presses. Their experiments comprised an agency task and a recognition task. In the agency task, the participants moved a box on the screen by pressing the up or down arrow key. Presented with an emotionally neutral word on the box, the participants rated the SoA over the box’s movement. The experiment used the spatial (in)congruence of the box movement’s direction and the delays between the key press and the box movements (100 or 900 ms) to manipulate the degree of SoA. In the recognition task, the participants judged whether they saw the word items on the screen during the agency task (old) or not (new). The results showed that the words presented in conditions with a stronger SoA (i.e., spatially congruent and 100-ms-delayed box movement) had a higher hit rate than those in the condition with a weaker SoA (i.e., incongruent or 900-ms-delayed).

Hon and Yeo^14^ discussed two explanations for the improved recognition performance by SoA. The first is the self-reference effect, in which stimuli processed while referring to the self are recognized better than stimuli processed in terms of their morphology and phonology^15–17^. However, this effect can be observed for stimuli merely assigned to the self, even without self-referential processing^18,19^. A stimulus perceived as “a stimulus I initiated” can be assigned to oneself; thus, it is possible that having an SoA over an outcome improves memory by making the outcome self-relevant. The second explanation is that additional retrieval cues are known to improve memory performance^20^. Therefore, the SoA tagged to action outcome^21^ might provide information used as an additional cue for retrieval and improve recognition performance.

The difference in recognition performance between conditions with strong and weak SoAs^14^ was open to interpretation. It remains unclear whether recognition improves in conditions with a stronger SoA or declines in conditions with a weaker SoA. This would require a comparison with a baseline condition where participants passively observe a stimulus without taking action and thus would not experience SoA over the stimulus. Given the action-induced memory enhancement^4^, recognition performance in a baseline condition without action should be lower than in conditions with action, regardless of the degree of SoA.

Moreover, it remains unclear whether the two recognition memory processes are modulated differently by the SoA. Recognition memory consists of *recollection* (i.e., a conscious and detailed memory of stimuli and events and the context in which they were learned) and *familiarity* (i.e., a feeling that stimuli and events are in memory although they are not specifically recalled)^22^. Previous studies on SoA and recognition memory^14,21^ have only employed old/new judgments in recognition tests and have not examined the influence of SoA on these two recognition processes. The remember/know (R/K) procedure examines the two processes^23,24^. Remember (R) and know (K) judgments are thought to reflect recollection and familiarity, respectively. Self-reference effects are found mainly in R judgments^25^, even if encoded items are merely assigned to oneself^26^. These results suggest that if the SoA improves recognition performance by making stimuli more self-relevant, it would also promote the recollection process and increase R judgments.

This study had two aims. The first was to investigate whether SoA improved recognition memory or weaker SoA disrupted recognition memory. We hypothesized that recognition performance would improve when the participants performed an action, and there was a strong SoA, than when they did not perform an action, or there was a weak SoA. The second aim was to investigate the influence of the SoA on the two processes of recognition memory. We hypothesized that recollection would be enhanced when the participants performed actions with a strong SoA.

Our experiment comprised two tasks. In the agency task, the participants moved a box on the screen by voluntary key presses, learned a word on the screen, and rated their SoA over the box movement. The box could move in a direction congruent or incongruent with the participants’ key presses. In the baseline condition, the box moved automatically. In the subsequent recognition task, the participants were tested for recognition memory for the word stimuli using the R/K procedure. We expected the SoA rating score and the recognition performance to be lower in the congruent, incongruent, and baseline conditions in that order. We also expected a larger number of R judgments (i.e., enhanced recollection) in the congruent condition than in the other conditions.

## Experiment 1

### Methods

#### Participants

An a priori power analysis using G*Power 3.1.9.6^27^ showed that 28 participants were required when we assumed a moderate effect (*f* = .25) for a three-level within-participant factor in repeated measures analysis of variance (rmANOVA) with an alpha of .05 and a statistical power of .80.

The participants were 31 female university students. The exclusion criteria were as follows: mother tongue was two or more; the proportion of key presses in the baseline condition (*M* = .02, *SD* = .04, range = .00–.19) was higher than .10; and the proportion of pressing the up arrow key (*M* = .53, *SD* = .11, range = .33–1.00) was higher than .80 or less than .20. Three participants, who met at least one of these criteria, were excluded from the analysis. The remaining 28 participants were analyzed (mean age = 22.14, *SD* = 6.50, range = 18–54). We used the Flinders Handedness Survey^28,29^ to determine the participants’ handedness and found that 25 were right-handed (*M* = 9.60, *SD* = 0.87), and three were left-handed (*M* = − 10.00, *SD* = 0.00). All the participants reported having normal or corrected-to-normal vision and no color blindness.

All participants in Experiments 1 and 2 provided written informed consent prior to the experiment. Experiments 1 and 2 were conducted in accordance with the Declaration of Helsinki and approved by the Humanities and Social Sciences Research Ethics Committee of Ochanomizu University (approval number 2021-137).

#### Apparatus

Participants were tested individually in a well-lit room. The stimuli were displayed on a 24-inch LCD monitor (PL2483H, Iiyama) with a viewing distance fixed at 57.3 cm using a chin rest. The participants responded using a QWERTY keyboard (R2S-JP4-BK, Topre). Stimulus presentation and response collection were controlled using PsychoPy 2021.2^30^ running on Windows 10.

#### Materials

We retrieved 103 words from a dataset of 121 two-character Japanese kanji compounds with neutral emotional valences^31^. We removed the five most-frequently-used and the four least-frequently-used words, making the entire set 112 words. We then removed the four words whose meanings were the most difficult to imagine and the five whose meanings were easiest to imagine, leaving us with 103 words. Finally, we randomly chose 94 words from those 103. We used 63 for the agency task, randomly assigning 21 of the 63 words to each of the three conditions. We used the remaining 31 words as new words in the practice and main trials of the recognition task. In the practice trials, we presented one word from each condition of the agency task and one new word. In the main trial, we presented ten words from each condition of the agency task and 30 new words.

#### Procedures

The participants completed the modified versions of the agency and recognition tasks in Experiment 2 of Hon and Yeo^14^.

**Figure 1:**
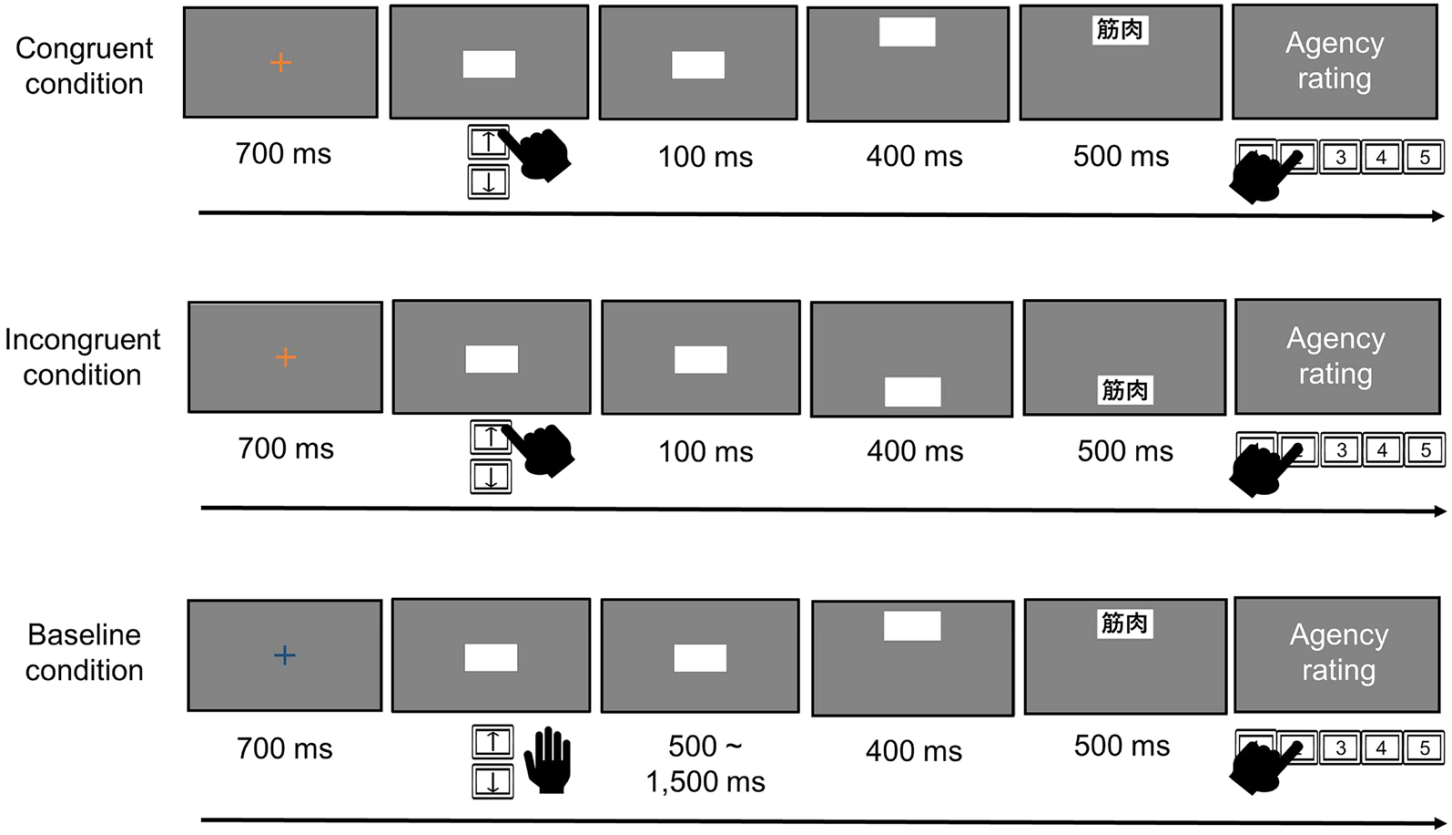
Schematic illustration of the agency task in Experiment 1.

At the beginning of each trial in the agency task (Fig. 1), we presented a fixation cross (height = 1.5°) at the center of the screen for 700 ms, followed by a white box (height = 4.0°, width = 8.0°). The participants freely chose and voluntarily pressed the up or down arrow key at their own pace using their right index fingers. The participants were instructed to avoid bias in their key selections. The box moved 5.14° upward or downward 100 ms after the key press. The pressed key and box movement orientations were consistent in the congruent condition but opposite in the incongruent condition. In the baseline condition, the box automatically moved upward or downward *without* the participant’s key press 500–1500 ms after the box appeared (randomly jittered in 250 ms steps). The direction of box movement in the baseline condition was random. The color of the fixation cross (orange or blue) instructed the participants to press the key. The correspondence between the color and conditions was counterbalanced between the participants. In any condition, 400 ms after the box’s movement, a word in black MS UI Gothic font (height = 1.0°, width = approximately 2.0°) was superimposed on the box for 500 ms. Finally, the participants rated their SoA over the box movement by answering the question “To what extent do you feel you controlled the movement of the box?” using a five-point Likert scale ranging from 1 (no control) to 5 (full control). The inter-trial interval was 700 ms.

In the recognition task, we presented a white fixation cross at the center of the screen for 500 ms, then replaced it with an old or new word on a white box. The word and box remained visible until the participants made an R, K, or New judgment for the presented word, as follows. When the participants felt they could remember the details of when they saw the word in the agency task (e.g., what they thought at the time), they pressed the C key to make an R judgment. When the participants could not remember the details but only knew they had seen the word in the agency task, they pressed the B key to make a K judgment. If participants felt they had not seen the word before, they pressed the M key to make a New judgment.

The participants completed three practice trials for each condition in the agency task. A word stimulus used for practice trials was “単語” (“word” in Japanese). To prompt intentional learning, the experimenter told the participants that they would be tested for recognition memory after the agency task. The participants completed 63 trials in the agency task. Immediately after the agency task, they completed practice trials for the recognition task, during which they were instructed on the meaning of R/K judgments and judged three old words and one new word. At the end of the practice, we checked to make sure the participants understood the R/K judgments and repeated the instructions as required. The participants then completed 60 trials in the recognition task. Finally, the participants completed a demographic questionnaire and handedness assessment. The average duration of the experiment was approximately 20 min.

#### Data analysis

The agency task trials, where participants pressed the up or down arrow key in the baseline condition, were excluded from analysis. The recognition task trials, where we presented words in the excluded agency task trials, were also excluded. We excluded 13 trials in total (0.38% of all trials, including agency and recognition tasks).

To check whether the intensity of SoA was manipulated, we conducted an rmANOVA with the Condition (congruent, incongruent, or baseline) as a within-participant factor on the individual mean SoA rating score. When the sphericity assumption was violated, the degree of freedom was corrected using the Greenhouse–Geisser method. Multiple comparisons in post-hoc tests were corrected using the Holm method.

For each participant, we then calculated the proportion of correct R/K judgments for old words in each condition, as well as the proportion of incorrect R/K judgments for new words. To determine whether the spatial congruence between the action and its outcome facilitated recollection and familiarity (i.e., R and K) in recognition memory, we conducted an rmANOVA with the Condition factor on the R- and K-recognition performances separately. Because the proportions of R and K were not independent, we performed rmANOVAs separately. The R-recognition performance was calculated by subtracting the proportion of R judgments for new words (i.e., false alarms) from that of R judgments for old words learned in each condition. A higher R-recognition performance indicates that the participants could better recall old words and more easily judge new words as new. The K-recognition performance was calculated using the same method.

When we did not find statistically significant main effects in the rmANOVA, we quantified the strength of evidence for null effects by calculating the exclusion Bayes factor (BF_excl_) across all models. We interpreted BF_excl_ values larger than 3 or 10 as moderate or strong evidence for null main effects of the factor and values between 1 and 3 as anecdotal evidence for null main effects, respectively^32^.

Data preprocessing was performed using R 4.2.0^33^. Statistical analyses were performed using JASP 0.16.3^34^ except that the bias-corrected and accelerated 95% confidence interval (CI) of *η*_g_^2^ with 2000 bootstrapping samples was calculated using the anovakun R function 4.8.6^35^.

### Results

#### Sense of agency rating

**Figure 2:**
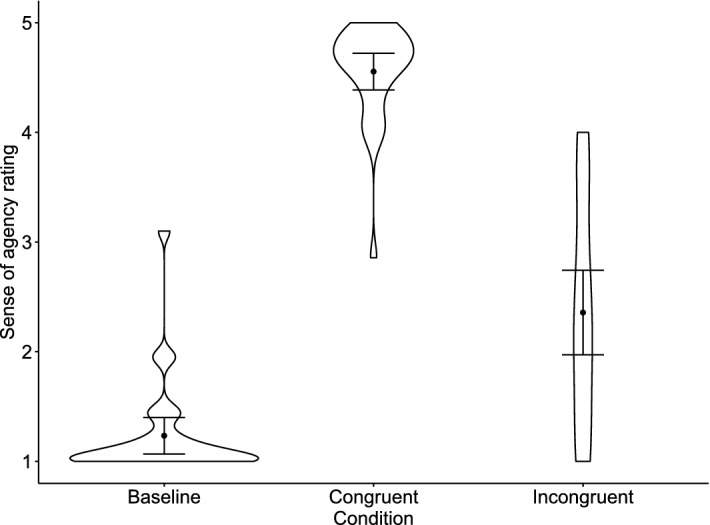
Sense of agency rating score in Experiment 1. Black dots depict means. Error bars depict 95% confidence intervals. Violin plots show the data distribution.

Mean SoA rating scores were 1.24 (*SD* = 0.45) for the baseline condition, 4.55 (0.45) for the congruent condition, and 2.36 (1.04) for the incongruent condition (Fig. 2). An rmANOVA revealed a significant main effect of the Condition (*F*(1.52, 40.98) = 184.01, *p* < .001, *η*_g_^2^ = .799, 95% CI [.732, .844]). Post-hoc tests showed significant differences in the SoA ratings between all conditions. Specifically, the SoA ratings were lower in the baseline condition than in the congruent (*t*(27) = − 18.86, *p* < .001, *d* = − 4.71, 95% CI [− 6.39, − 3.03]) and incongruent conditions (*t*(27) =  − 6.38, *p* < .001, *d* = − 1.59 [− 2.40, − 0.78]). The difference between the congruent and incongruent conditions was also significant (*t*(27) = 12.48, *p* < .001, *d* = 3.12, 95% CI [1.91, 4.32]). These results suggested that the degree of SoA was successfully manipulated in this experimental paradigm.

#### Remember/Know recognition performance

**Figure 3:**
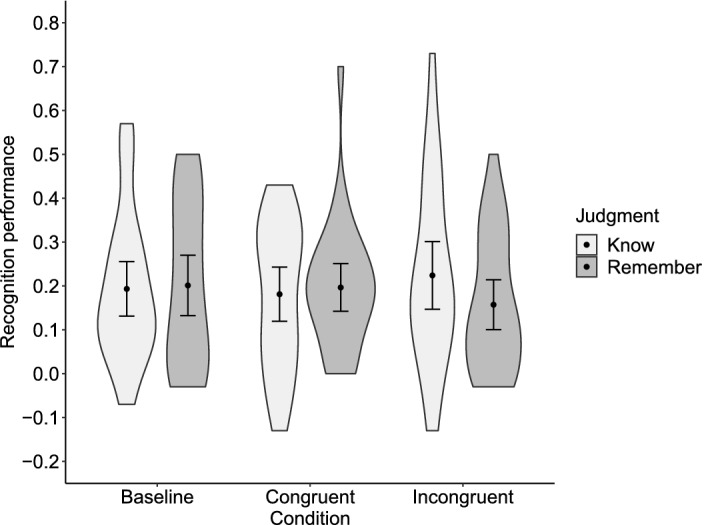
Recognition performance in Experiment 1. The Remember-recognition performance was calculated by subtracting the proportion of Remember judgments for new items from that for old items learned in each condition. The Know-recognition performance was calculated in the same manner. Black dots depict means. Error bars depict 95% confidence intervals. Violin plots show the data distribution.

Table 1 shows the proportions of R and K judgments for old words in each condition and new words. The R-recognition performance was .201 (*SD* = .186) in the baseline condition, .196 (.147) in the congruent condition, and .157 (.154) in the incongruent condition (Fig. 3). The K-recognition performance was .193 (*SD* = .168) in the baseline condition, .181 (.167) in the congruent condition, and .224 (.209) in the incongruent condition. The rmANOVA revealed no significant main effect of Condition on R-recognition performance (*F*(2, 54) = 1.64, *p* = .204, *η*_g_^2^ = .015, 95% CI [.000, .051]) or K-recognition performance (*F*(2, 54) = 0.50, *p* = .608, *η*_g_^2^ = .010 [.000, .045]). We found anecdotal evidence for the null effect of Condition on R-recognition performance (BF_excl_ = 2.71), and moderate evidence for the null effect on K-recognition performance (BF_excl_ = 6.24).

**Table 1:** Mean proportion of Remember and Know judgments for old items in each condition and for new items during the recognition task in Experiment 1.

	Remember judgment	Know judgment
Baseline condition	.219 (.196)	.383 (.165)
Congruent condition	.214 (.151)	.371 (.209)
Incongruent condition	.175 (.155)	.414 (.212)
New items	.018 (.024)	.190 (.101)

SD in parentheses.

### Discussion

This experiment tested whether recognition performance and SoA were modulated by spatial congruence between motor actions and their visual outcomes. We also examined the different effects on the two recognition processes (i.e., recollection and familiarity). We found that the SoA score was higher when the participants received spatially congruent action outcomes than when they received incongruent outcomes or did not take action. This is consistent with the previous finding that SoA requires voluntary action and is decreased by spatially incongruent outcomes^11,12^. Contrary to our hypothesis, we found evidence for the lack of influence of voluntary action and action–outcome congruence on R- and K-recognition performances.

Our results are inconsistent with Hon and Yeo^14^, who reported that the hit rate in a recognition task was higher in conditions where participants experienced a stronger SoA. This inconsistency might be due to differences in the participants’ learning strategy. The participants in Hon and Yeo’s^14^ study performed incidental learning of words without prior instruction on the task requirements. In contrast, our experiment employed intentional learning; therefore, we instructed the participants to make an effort to memorize the words. We chose intentional learning to reduce random errors because some participants in our pilot experiment noticed that their memory for the words viewed in the agency task would be tested later while others did not. Therefore, intentional learning might have enhanced recognition memory even in the incongruent and baseline conditions and hindered the effects of action–outcome congruence and voluntary action on recognition performance.

Another potential explanation is that the participants’ SoA over the appearance of the words was not sufficiently strong to enhance recognition performance. Given that SoA is likely to emerge for a temporally proximal action outcome^11–13^, the participants’ key presses might have provided a sufficiently strong SoA over the box movement 100 ms after the key press. However, the SoA might have decreased for words presented 400 ms after the box movement. When a key press causes two successive sensory outcomes, the SoA over the second outcome can be weaker than for the first^36^. Therefore, we further hypothesized that when a key press directly initiated the appearance of a word, a sufficient degree of SoA over the appearance of the word would emerge, enhancing recognition memory for the words.

In Experiment 2, we employed incidental learning in the recognition task and key-press action to initiate the appearance of a word in the agency task to examine the possibility that learning strategies and potentially weakened SoA could have hindered the memory enhancement found in the previous study^14^.

## Experiment 2

### Methods

#### Participants

The participants were 28 female students who did not meet the exclusion criteria for Experiment 1 (mean age = 20.18, *SD* = 2.75, range = 17–28). Twenty-six were right-handed (mean Flinders Handedness Survey score = 9.65, *SD* = 0.89) and two were mixed-handed (scores of − 2 and 0).

#### Apparatus

Identical to Experiment 1.

#### Materials

We used the same 103 words used in Experiment 1. Nine and 63 words were randomly selected from of the stimulus set for the practice and main trials of the agency task, respectively. One and 30 words were used as new words in the practice and main trials of the recognition task, respectively.

#### Procedures

**Figure 4:**
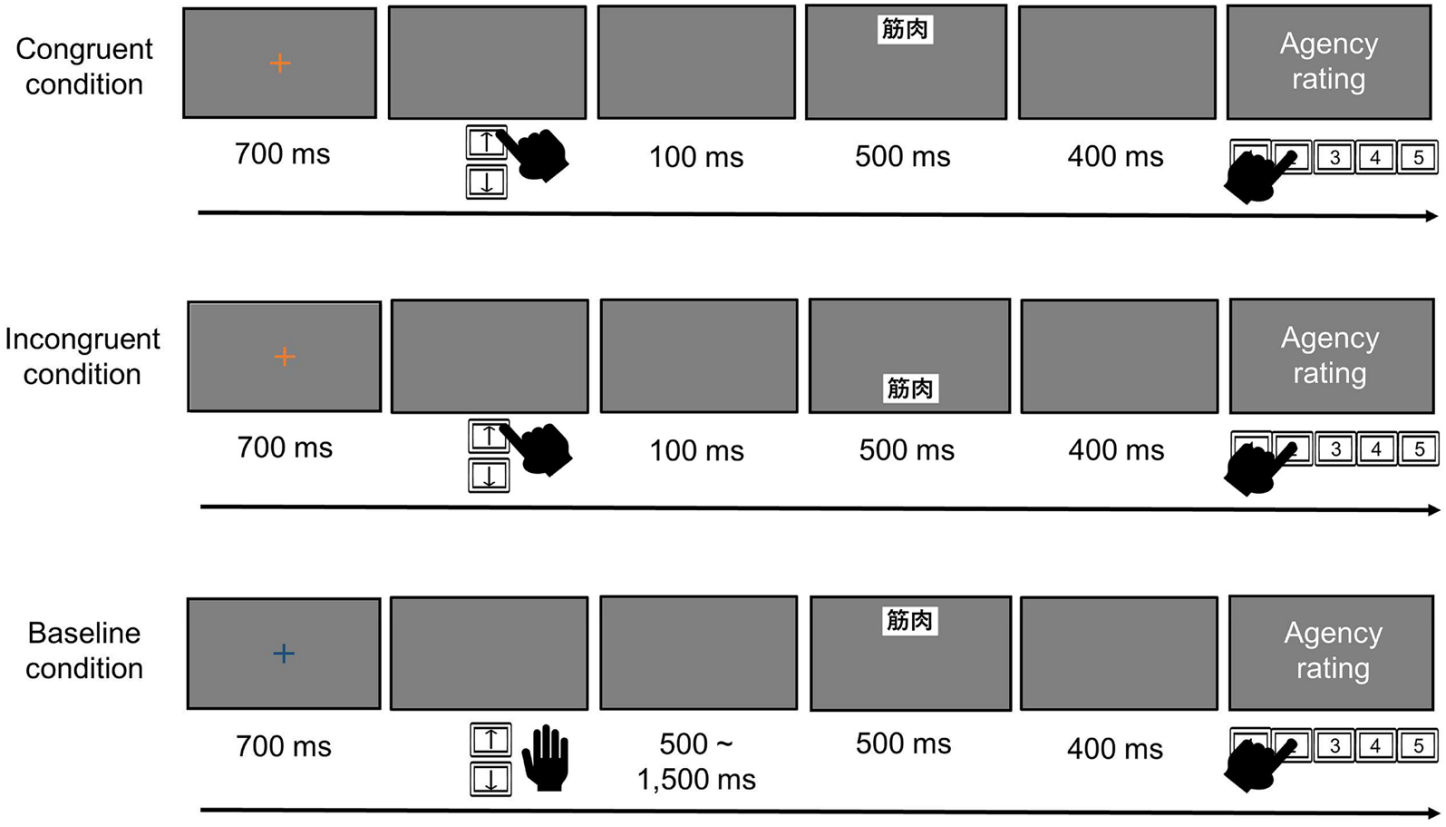
Schematic illustration of the agency task in Experiment 2.

The tasks were identical to those in Experiment 1 except for the following. In the agency task (Fig. 4), a blank screen was presented following the fixation cross. In the congruent and incongruent conditions, a word on the box was presented for 500 ms upward or downward from the center of the screen 100 ms after the participant’s key press. In the baseline condition, the word was automatically presented for 500 ms upward or downward 500–1500 ms after the blank screen. Following the presentation of the blank screen for 400 ms, the participants answered the question “To what extent do you feel you controlled the appearance of the word?” The experimenter did not tell the participants that they would perform the recognition task until after they had completed the agency task.

#### Data analysis

Identical to Experiment 1. We excluded 17 trials (0.49%) from the analyses.

### Results

#### Sense of agency rating

**Figure 5:**
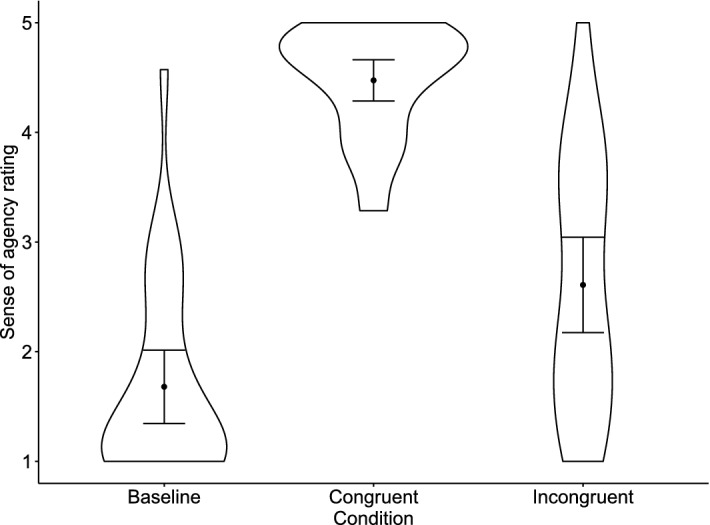
Sense of agency rating score in Experiment 2. Black dots depict means. Error bars depict 95% confidence intervals. Violin plots show the data distribution.

The SoA rating scores were 1.68 (*SD* = 0.90) for the baseline condition, 4.47 (0.51) for the congruent condition, and 2.61 (1.18) for the incongruent condition (Fig. 5). An rmANOVA revealed a significant main effect of Condition (*F*(2, 54) = 100.15, *p* < .001, *η*_g_^2^ = .631, 95% CI [.462, .724]). Post-hoc tests showed that the SoA score was significantly lower in the baseline condition than in the congruent (*t*(27) =  − 13.89, *p* < .001, *d* = − 3.09, 95% CI [− 4.26, − 1.92]) and incongruent conditions (*t*(27) =  − 4.61, *p* < .001, *d* = − 1.03 [− 1.67, − 0.38]). The SoA score was significantly higher in the congruent condition than in the incongruent condition (*t*(27) = 9.28, *p* < .001, *d* = 2.06, 95% CI [1.19, 2.94]).

#### Remember/Know recognition performance

**Figure 6:**
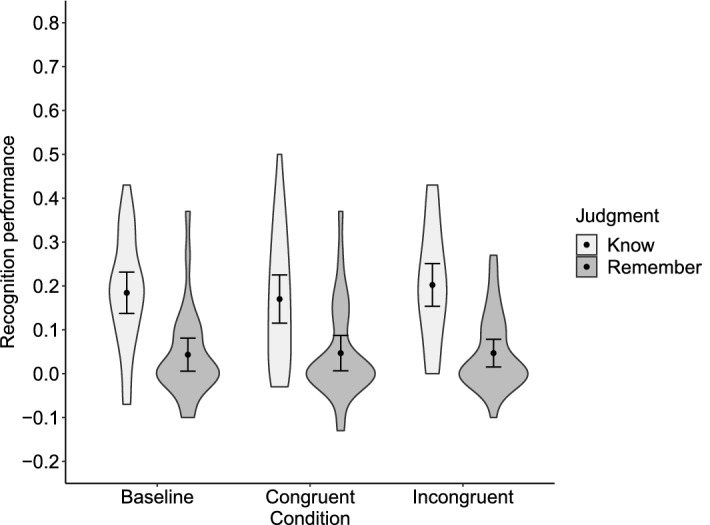
Remember- and Know-recognition performances in Experiment 2. Black dots depict means. Error bars depict 95% confidence intervals. Violin plots show the data distribution.

Table 2 shows the proportions of R and K judgments for old words in each condition and new words. R-recognition performance was .043 (*SD* = .102) in the baseline condition, .047 (.109) in the congruent condition, and .047 (.085) in the incongruent condition. K-recognition performance was .184 (*SD* = .127) in the baseline condition, .170 (.148) in the congruent condition, and .202 (.131) in the incongruent condition (Fig. 6). The rmANOVA revealed no significant main effect of Condition on R-recognition performance (*F*(2, 54) = 0.02, *p* = .984, *η*_g_^2^ < .001, 95% CI [.000, .000]) or K-recognition performance (*F*(2, 54) = 0.49, *p* = .613, *η*_g_^2^ = .010 [.000, .038]). We observed moderate evidence for null effects of Condition on R-recognition performance (BF_excl_ = 9.38) and K-recognition performance (BF_excl_ = 6.42).

**Table 2:** Mean proportion of Remember and Know judgments for old items in each condition and for new items during the recognition task in Experiment 2.

	Remember judgment	Know judgment
Baseline condition	.079 (.113)	.371 (.169)
Congruent condition	.082 (.125)	.357 (.199)
Incongruent condition	.082 (.116)	.389 (.215)
New items	.035 (.051)	.187 (.138)

SD in parentheses.

### Discussion

Experiment 2 re-examined the effect of SoA on recollection and familiarity in recognition memory by employing incidental learning and making the key press action and its outcome (i.e., words) temporally closer. However, as in Experiment 1, we found no effects of voluntary action and action–outcome spatial congruence on recollection and familiarity, although the SoA over word appearance was successfully manipulated. These results suggest that voluntary action and action–outcome spatial congruence and the associated SoA do not modulate recognition memory for the outcome, even during incidental learning.

#### General discussion

This study examined whether voluntary action with stronger SoA improved recognition memory or that with weaker SoA disrupted it. It also tested whether SoA enhanced the recollection process more than the familiarity process in recognition memory. The participants voluntarily pressed a key, initiating a box movement preceding the appearance of the word to be memorized (Experiment 1) or the word appearance per se (Experiment 2), followed by a recognition task using the R/K procedure. In both experiments, SoA, measured by a subjective rating, was successfully manipulated by voluntary action and spatial congruence between the key press and its visual outcome. However, we found evidence for the lack of effects of voluntary action and action–outcome congruence on R- and K-recognition performances, regardless of intentional (Experiment 1) or incidental learning (Experiment 2). These results are inconsistent with the findings presented by Hon and Yeo^14^, who suggested that SoA can improve recognition memory.

There could be two explanations for this discrepancy. First, we might have failed to manipulate the participants’ SoA. However, we can rule this out because their SoA was consistently and robustly manipulated in our experiments. The SoA rating scores were higher when the participants pressed keys than when they did not. Furthermore, the SoA was stronger when the outcome was spatially congruent with the direction of the key-press action. These results are consistent with previous results suggesting that SoA is manipulated by spatial congruence between action and its visual outcome^11,12^ and also reflect a model^10^ postulating that SoA results from a match between motor-based predictions and actual sensory outcomes. Second, given that a larger temporal interval between action and outcome decreases SoA^11–13^, the temporal interval between the key presses and the word presentation might have disrupted or nullified the association between the SoA and recognition memory. However, we can also rule out this possibility because the intervals of 500 ms in Experiment 1 and 100 ms in Experiment 2 are the same as or shorter than the small delay condition in Hon and Yeo^14^.

We assumed three potential mechanisms for memory enhancement by the SoA. The first is the self-reference effect, where information associated with the self is more likely to be remembered^18,19^. Given that the self-reference effect has been known to influence the recollection process of recognition^24,25^, we could predict that SoA will make the action outcome self-relevant and enhance the recollection process. However, our results suggested no enhancement in the recollection (or familiarity) process. Second, the SoA can be tagged to the action outcome^21^ (i.e., word) and provide additional cues to enhance memory retrieval^20^. However, we found no differences in recognition performance between conditions in either intentional (Experiment 1) or incidental learning (Experiment 2). Third, attention allocated to stimuli that elicit an SoA^37,38^ may explain memory enhancement. As stimuli attracting more attention are recognized better than those that attract less attention^39^, stimuli with a stronger SoA may result in improved recognition performance. However, our data do not allow for the analysis of the effect of attention, and future studies should investigate memory and attentional benefits induced by SoA.

We found no difference in recognition performance between the conditions with (i.e., congruent and incongruent) or without voluntary action (i.e., baseline). This was inconsistent with previous findings that recognition performance for visual stimuli initiated by or coinciding with a key press was higher than for stimuli without a key press^4,5^. The learned stimuli were presented approximately 0 ms^﻿5^ or 200 ms^4^ after the key press in previous studies, whereas in Experiment 2, the delay between the key press and the presentation of the learned stimulus was 100 ms, which falls within the time window to induce memory enhancement (0–200 ms). Thus, it is unlikely that the different time intervals between the action and presentation of the learned stimulus were responsible for the lack of memory enhancement in our study.

We propose three potential explanations for this discrepancy with the results of previous studies. First, as Kinder and Buss^6^ and Shimane et al.^7^ have suggested, action may not have a direct impact on recognition memory. Instead, motor engagement leading to motor execution or inhibition can enhance recognition memory. In our study, participants randomly engaged in motor execution or inhibition, with trials requiring either voluntary action or no movement being cued by the color of the fixation cross in a single session. Therefore, recognition memory might have been enhanced even in our baseline condition without voluntary action, and the difference between the conditions was apparently nullified.

The second explanation is the visual stimulus. A previous study^4^ used object photographs as stimuli and found memory enhancement by simple key-press actions, whereas we used two-compound Japanese kanji words, failing to find any effects. Studies have suggested that pictures are better recognized than words, even when representing the same object (i.e., picture superiority effect^40,41^). Given this effect, the word stimuli in our experiments may not have been sufficient to detect the effect of voluntary action on memory enhancement.

The third explanation is the difference in participants’ arousal levels. An inverse U-shaped correlation has been suggested between cognitive performance and arousal in complex tasks, such as memory formation^42–44^. Indeed, Yebra et al.^4^ found that participants with high arousal levels during Go responses did not exhibit enhanced memory performance for Go items. This result suggests that in some individuals, Go responses might cause the release of noradrenaline beyond the optimal amount for encoding, disrupting the encoding of Go items^4^. The participants in our study who did not show enhanced recognition performance in the conditions with key presses could have had higher-than-optimal arousal levels. Therefore, future studies should include determining whether the participants’ arousal is elevated beyond optimal levels and identifying the causes of the elevation.

### Limitations

Our study had three limitations. First, we assessed the participants’ SoA by self-report. Thus, it is possible that the measured SoA was biased by demand characteristics. It might be beneficial to employ an implicit measure of SoA, such as intentional binding^45^ to reduce demand characteristics. Future research should also examine the relationship between memory and SoA as measured by intentional binding. Second, our null findings were limited to the memory of verbal stimuli during a key press action task. Sugimori and Asai^21^ employed a task in which participants performed continuous movements to make various hand postures (e.g., rock, paper, scissors) while observing video feedback of their hands. They suggested that recognition memory for the hand postures was enhanced by SoA. To generalize our findings, future studies should also use nonverbal stimuli such as object images^4,19^ and other motor tasks (i.e., continuous action^21^). Finally, our study did not directly replicate the experiments by Hon and Yeo^14^; thus, we cannot conclude that SoA does not improve recognition memory. The present and previous studies had methodological differences, such as learning strategies and SoA manipulation (e.g., varying action-outcome delays). These differences may mask the effects of SoA on memory.

### Conclusions

This study investigated the effects of an SoA on the recollection and familiarity of recognition using the R/K procedure. Contrary to our hypotheses, our results from two experiments suggested that neither spatial congruence between a key press action and its verbal visual outcome nor the inclusion of an action modulated the performances of recollection and familiarity in recognition memory, regardless of the latency of word presentation and learning strategies. These results were also inconsistent with previous findings that memory can be enhanced by stronger SoA^14^ and voluntary action^4,5^. To further elucidate the relationship between SoA and memory, future replication studies examining methodological concerns (e.g., measures, stimuli, and motor tasks) and meta-analytic studies are needed.

## Data Availability

The word stimuli and raw data associated with Experiments 1 and 2 are publicly available at https://osf.io/bwhvp/.
